# Phasing out of the Universal Mega Dose of Vitamin-A Prophylaxis to Avoid Toxicity

**DOI:** 10.3934/publichealth.2017.1.38

**Published:** 2017-01-20

**Authors:** Sudip Bhattacharya, Amarjeet Singh

**Affiliations:** Department of Community Medicine, School of Public Health, PGIMER, Chandigarh, India

**Keywords:** keratomalacia, Vitamin-A toxicity, mortality

## Abstract

Childhood blindness due to corneal ulceration was prevalent among poor Indian children. To tackle this situation, the National Institute of Nutrition (NIN), Hyderabad, India, Vitamin-A (Vit-A) prophylaxis programme was launched nationally in 1970 after field testing. Research of Indian Council for Medical Research (ICMR) documented that prevalence of Vit-A deficiency signs such as Bitot's spot decreased among children, over a period of time. However, this decrease cannot be ascertained is due to mass Vit-A prophylaxis programme. This is because coverage was low and patchy. Improved nutrition status, wider vaccination coverage, increased rate in breast feeding and improvement of healthcare services played a crucial role. Rather many studies revealed that (mass prophylaxis to the child who is having adequate Vit-A level) it may be harmful to certain group of children as a result of acute toxic symptoms. High dose of Vit-A is capable of loss of bone density-hence retarded growth may be observed in susceptible individuals. To tackle this issue food based approach should be promoted (which includes breast feeding) along with timely measles vaccination. The children who have signs of Vit-A deficiency (e.g. night blindness, xeropthalmia, Bitot's spot) or post measles children should receive Vit-A in age specific daily doses for two weeks along with Vit-A rich food, like green leafy vegetables, red palm oil, liver etc. Public spirited citizens, together with scientific community in India, should discourage this “one size fit to all” approach. It will not only avoid the ill effects of high dose of Vit-A but also it will help us optimal utilization of health resources in a resource poor country like India.

## 1. Introduction

International public health policy makers recommend mega dose of Vitamin-A (Vit-A) to all children aged between 6–60 months irrespective of nutritional status. According them it is a major public health problem in 1950s–60s. In many countries the mega dose of Vit-A was given over enthusiastically to the children. Even today it is still continuing as a part of routine supplementation irrespective of areas where Vit-A deficiency signs are rare [1].

At that point of time lot of promotion (hype) was done for synthetic Vit-A supplementation by international communities. They claimed that Vit-A prophylaxis has multiple benefits. It can reduce diarrheal death, acute respiratory infections, mortality and improves immunity [2].

In India keratomalacia and kwashiorkor was emerged as important public health issue 1950 to 1960. Leading cause of childhood blindness (ocular morbidity) due to corneal ulceration was rampant among poor children. To overcome this situation, in India, the National Institute of Nutrition (NIN), pilot tested with mega dose of Vit-A to the vulnerable children so that the problem of keratomalacia may be controlled. It was the basis of launching mega dose of Vit-A among children in India [3]. The problem starts in 2001, more than 30 children was died after administration of mega dose of Vit-A in Nepal, India. A debate was initiated scientific community regarding mega dose prophylaxis of Vit-A. The scientific community was divided in to two parts [4].

This review article challenges the appropriateness of administering mega dose of Vit-A to all children (including healthy) between 6–60 months. Approximately half billion capsules are manufactured on yearly basis to distribute among 200 million children across 100 countries [1]. We can say that it is just a nexus of business personnel, scientific community and big agencies. It has been observed that international funded programmes are not always driven by science. In any politically influenced policy, scientific evidence plays a very little role. It can be manipulated easily by policy makers with the help of scientific communities and business personnel. Most of the policies are driven by money. For an example US Agency for International Development (USAID) provides one of the biggest overseas developmental assistance for Vit-A prophylaxis programme. Although the grant helped to the recipient countries yet it served more the interest of United States [1].

The second driving force for promotion of Vit-A capsules, is a big pharmaceutical industry, which is known as “invisible hands” of markets. Last but not the least we cannot ignore the influence of culture. Due to lack of grass root level experience of donor (rich) countries they usually have failed to assess the need of the recipient (poor) countries [1].

Several studies have documented (Table 1, 2) about the poor association between of mega dose of Vit-A supplementation and reduction in childhood mortality. Which was one of the major reason for launching Vit-A prophylaxis programme. During impact assessment for mass Vit-A administration by National Institute of Nutrition (NIN), Hyderabad, India, it was found that the coverage was low and patchy among children. One of the reasons for poor coverage may be constraint in resources. As poor country like India there are multiple competing priorities for resources, as resources are scarce. Even the justification of giving mega dose is debatable, even in Vit-A deficient child. That study concluded that mega dose of Vit-A supplementation has no role in reduction on child hood mortality and morbidity rates [3]. The Similar findings were observed in Assam and (study conducted from Johns Hopkins University) and in Uttar Pradesh [5],[6].

Another study reported from Christian Medical college, Vellore, that there was quick decline in serum Vit-A (within 3 weeks) levels after administering mega dose, in some of the cases. Hence the effectiveness of mega dose remained debatable [7].

Later it was observed in a study carried by Harvard University that mega dose of Vit-A failed to resolve few cases of Bitot's spots. Quick fall in the serum level may be the reason, as illustrated in previous study [1].

Surveys in 1980s in India were revealed that keratomalacia was no more considered as major public health problem. Vit-A deficiency was seen in only in poor community in the form of Bitot's spot and mild form of chronic Vit-A deficiency. According to ICMR data, current prevalence of Bitot's spots in India is 0.3–0.7 percent and a gradual decline was noticed. It was due to overall development in health system and improvement in nutrition status not due to mega dose supplementation. Various studies revealed that mass administration (even in healthy children) of mega dose of Vit-A was not needed. They also considered the possible harmful effects of mega dose Vit-A supplementation. It was opined that it should be phased out [8].

## 2. Massive Doses Prophylaxis and Child Hood Mortality

The previous claim by 30% reduction of child hood mortality by mega dose administration was questionable [1]. The study of John Hopkins group in Indonesia claimed reducing mortality by administration of mega dose of Vit-A, suffered from many methodological issues. There were issues like non randomization of baseline samples, control group had disease (malnutrition along with signs of Vit-A deficiency), cause of death was not mentioned and randomization done at village level and children data was presented [1].

Field trials, who favored mega dose supplementation, appeared to be biased. Those trials were conducted in areas where clinical deficiencies were common along with poor health facilities.

**Table 1.** Summary of Studies recommended for phasing out of mega dose of Vit-A supplementation.

Published article/commentary	Conclusions
Latham M. The great vitamin A fiasco. *World Nutrition* May 2010; 1, 1: 12–45.	Megadose of Vitamin-A shall be scrapped [1].
Gopalan, Sachdev, Kapil, Soekirman et al. Responses to The great vitamin A fiasco. World Nutrition June 2010; 1, 2: 78–119.	Mega dose do harm, Food-based approaches are best [3].
Gopalan, Sachdev, Kapil, Soekirman et al. Responses to The great vitamin A fiasco. World Nutrition June 2010; 1, 2: 78–119.	Time to phase out the universal Vitamin-A supplementation programme [3].
Gopalan, Sachdev, Kapil, Soekirman et al. Responses to The great vitamin A fiasco. World Nutrition June 2010; 1, 2: 78–119.	Vitamin-A capsules have been the major focus of the Vitamin-A deficiency eradication programme in Indonesia. But other programmes, such as fortification, are crucial. Public health interventions in general still face a lot of challenges in Indonesia. Slowly we are addressing many of the underlying factors that affect Vitamin-A status, such as breastfeeding, home gardens, water supply, sanitation, immunisation, and health education [3].
Nesheim M. Need for long-term benefits. [Letter] World Nutrition, June 2010, 1, 2: 106.	Support for local agriculture, and for health and sanitation initiatives, are likely to provide the long-term health benefits [9].
Reddy V. Need for food-based programmes. [Letter] World Nutrition, June 2010, 1, 2: 106–107.	Vitamin-A may have the potential to avert deaths in children, as shown in some of the controlled trials with adequate coverage. But the mortality impact has not been demonstrated in populations where the Vitamin-A programme has been in operation for several years, since the children who are at greatest risk are often inaccessible. The wisdom and validity of the current practice of giving large doses of Vitamin-A to young children has also been questioned [10].
Lyons G. Need to go and stay local. [Letter] World Nutrition, June 2010, 1, 2: 112–113.	In the 1970s there was a measles outbreak in North Malaita where children were going blind not just because of Vitamin-A deficiency, but because of the combination of deficiency and measles. Dr Latham's excellent, critical commentary provides further justification of the “Go Local” approach to addressing malnutrition. When “donor fatigue” causes the likely demise of simplistic “medicinal dosing” strategies, let us hope that a concerted effort will be made to encourage and implement the food system approach [11].
Amdekar Y A et al. Vitamin Controversy. World Nutrition, June 2010, 1, 2: 114–116.	In summary, routine supplements of vitamins are unnecessary. It may be required for normal pre-term new-borns. Otherwise supplements of vitamins should be reserved for treatment of deficiency states. Those who need vitamin supplements often require therapeutic doses of vitamins to treat specific deficiencies, and are not benefited by routine doses [12].

**Table 2.** Summary of Studies Linking Vit-A with Reduction of Mortality and Morbidity.

Study and area	Sample			Intervention (Vitamin A)	Outcome measures	Overall Reduction	
	age (mo)	size	adequacy			mortality	morbidity
Rahamathullah et al. [13], Trichy, rural Tamil Nadu	6–60	15419	Not mentioned	8333 IU per week	mortality(include accidental deaths) morbidity (ARI, diarrhea); long recall period	yes	no
Vijayraghavan et al. [14] rural Hyderabad	12–60	15775	Not mentioned	2,00,000 IU 6 monthly, 2 doses	mortality (cause not ascertained), morbidity (ARI, diarrhea); severity not assessed	no	no
Kothari [15], urban slum, Mumbai	<12	387	Not mentioned	2,00,000 IU, doses? duration? frequency?	mortality (cause not ascertained), morbidity (not defined) follow up over 3 years	yes	no
Ramakrishnan et al. [16], rural Tamil Nadu	6–36	583	adequate to detect 25% reduction in morbidity	1,00,000 IU to <1 yr, 2,00,000 IU to > 1yr, 4 monthly for 1 yr	morbidity (ARI, diarrhea) defined and assessed for frequency and duration	-	no
Agarwal et al. [17], rural Varanasi	1–72	15247 And 2514	not mentioned	50,000 IU to <6 mo, 1,00,000 IU to >6 mo, 4 monthly for 1 yr	mortality (cause ascertained), morbidity (measles, ARI, Otitis media, skin infections)	yes?	yes
Bhandari et al. [18], urban slum, New Delhi	12–60	900	adequate to detect 25% reduction in diarrhea	2,00,000 IU single dose	morbidity (ARI, diarrhea) defined and assessed for 3 months after acute diarrheal episode	-	no
Dewan et al. [19], tertiary hospital, New Delhi	6–60	216	not mentioned	1,00,000 IU single dose	duration of acute diarrheal episode, no long term follow-up.	-	no
Venkatarao et al. [20], rural Tamil Nadu	Newly born and her mother	909 pairs	adequate to detect 10% reduction in ARI/diarrhea incidence	3,00,000 IU to mother and 2,00,000 IU to infant at 6 mo of age	morbidity (ARI, diarrhea) defined and assessed for incidence, severity and duration, till 1 yr of age	-	no
Coles et al. [21], rural Tamil Nadu	0–6	465	power to detect differences was low	7000 µg retinol, 2 doses within 48 h of birth	Nasopharyngeal pneumococcal carriage at 2,4,6 mo of age, mortality, morbidity not analyzed	-	-

It was also mentioned in Beaton report, that there was no conclusive evidence that the “magic bullet” (Vit-A capsule) can reduce mortality. This key comment in that report was deliberately ignored. The report also advocated for gradual and sustainable multi-pronged approaches for combatting Vit-A Deficiency (VAD), instead of “knee jerk response” as mega dose supplementation of Vit-A to all children [1].

In many countries like India, the rapid decline in Vit-A deficiency occurred due to overall improvement in health and other sectors. In 1970s immunization coverages improved from 5–7% to 60–90% considering state to state variability [8].

So our target specific approach is justifiable for multiple reasons. Firstly, the role of mortality reduction was debatable. Secondly the prevalence in keratomalacia has decreased. Thirdly we have limited resources with competing health priorities.

## 3. Massive Doses of Vit-A are Toxic

Current government health policy (Indian) advocates mega dose of Vit-A, according to policy a child (9^th^ to 36^th^ month ) have to receive 1,700,000 IU of Vit-A, it is massive [22],[23]. Massive dose can cause acute and chronic toxicity to certain group of children. Ranging from bulging of anterior fontanelle (sign of increased intracranial tension) to mental retardation, even death incidence [1]. Thus it can do more harm than good.

## 4. Vitamin-D and Zinc Antagonism

Study revealed that Vit-A can accelerate loss in bone density by the process of demineralization, followed by growth retardation. This issue can be further complexed by other sub issues like poor family, malnourished mother, underweight child, poor availability of sunlight in slum areas etc.

Vitamin-D deficient child may also suffer from zinc deficiency and other micronutrient deficiency, which also leads to growth retardation. By administrating mega dose of Vit-A it can be further aggravated. This is a serious issue; we have to look this aspect very seriously [3].

## 5. The Best Approach

A balanced diet is the ultimate way to prevent micronutrient deficiency. Government of India should promote production and consumption of locally available seasonal fruits and vegetables. Green leafy vegetables, animal liver, red palm oil are the best sources of for Vit-A. Promotion of kitchen, school and community gardening are very important, but it is usually ignored. We as Indians have tremendous opportunity to encash our natural resources, because most of our people are resending in villages where fresh vegetables and fruits are easily available. Another important aspect is that, garden foods and animal foods are natural, they are fresh and it cannot be substituted by any synthetic capsules (“magic bullets”). Gardening (by exercise) prevents us from chronic diseases also. So single health promotive approach may results in multiple health benefits.

For infant and children, nothing is better than breastmilk. It is a natural source of Vit-A, besides providing calorie, water and other protective factors (immunoglobulins). We have to promote increased breastfeeding rates among mothers [3].

Vegetables and fruits are not only good sources of Vit-A but several other micronutrients. A balanced diet that includes adequate amounts of variety of vegetables and other foods is the surest way of preventing micronutrient deficiencies. Another advantage of consuming Vit-A, available from natural resources i.e. pro-Vit-A carotenoids are adequately bioavailable and cause no harm (nontoxic) if it is taken in excessive amount is another important aspect [24].

As part of India's National Health Mission (NHM) and Integrated Child Development (ICD) programmes, post measles children and who have Bitot's spot should receive synthetic Vit-A in recommended daily doses (6–12 months is 600 IU/day; that for children between 4 and 5 years old is 900 IU/a day) for 14 days and concurrently culturally acceptable balanced diet(including green vegetables and fruits) should be encouraged [1],[23].

Overenthusiastic use of Vit-A supplements (under Universal Immunization Programme) to all the children (6–60 moths) should be phased out [23].

It is also leading to non-judicious utilization of our limited health resources which can be utilized in promotion of other public health activities. The children likely to suffer Vit-A deficiency can be identified and preventive/curative dose of Vit-A can be given. However, it is difficult to predict in a community which child may suffer from measles. Hence, Vit-A supplementation cannot be compared with measles immunization which is recommended universally for all children [23].

## 6. Conclusion

Public spirited citizens, together with scientific community in India, must ensure the phasing out of the universal mega-dose Vit-A prophylaxis approach.
